# Genetic influences on disease course and severity, 30 years after a clinically isolated syndrome

**DOI:** 10.1093/braincomms/fcad255

**Published:** 2023-10-04

**Authors:** Nitin Sahi, Lukas Haider, Karen Chung, Ferran Prados Carrasco, Baris Kanber, Rebecca Samson, Alan J Thompson, Claudia A M Gandini Wheeler-Kingshott, S Anand Trip, Wallace Brownlee, Olga Ciccarelli, Frederik Barkhof, Carmen Tur, Henry Houlden, Declan Chard

**Affiliations:** NMR Research Unit, Queen Square Multiple Sclerosis Centre, University College London Queen Square Institute of Neurology, London WC1N 3BG, UK; NMR Research Unit, Queen Square Multiple Sclerosis Centre, University College London Queen Square Institute of Neurology, London WC1N 3BG, UK; Department of Biomedical Imaging and Image Guided Therapy, Medical University Vienna, 1090 Vienna, Austria; NMR Research Unit, Queen Square Multiple Sclerosis Centre, University College London Queen Square Institute of Neurology, London WC1N 3BG, UK; NMR Research Unit, Queen Square Multiple Sclerosis Centre, University College London Queen Square Institute of Neurology, London WC1N 3BG, UK; Centre for Medical Image Computing (CMIC), Department of Medical Physics and Biomedical Engineering, University College London, London WC1E 6BT, UK; Universitat Oberta de Catalunya, 08018 Barcelona, Spain; NMR Research Unit, Queen Square Multiple Sclerosis Centre, University College London Queen Square Institute of Neurology, London WC1N 3BG, UK; Centre for Medical Image Computing (CMIC), Department of Medical Physics and Biomedical Engineering, University College London, London WC1E 6BT, UK; Department of Clinical and Experimental Epilepsy, University College London, London WC1N 3BG, UK; NMR Research Unit, Queen Square Multiple Sclerosis Centre, University College London Queen Square Institute of Neurology, London WC1N 3BG, UK; NMR Research Unit, Queen Square Multiple Sclerosis Centre, University College London Queen Square Institute of Neurology, London WC1N 3BG, UK; NMR Research Unit, Queen Square Multiple Sclerosis Centre, University College London Queen Square Institute of Neurology, London WC1N 3BG, UK; Department of Brain and Behavioural Sciences, University of Pavia, 27100 Pavia, Italy; Brain MRI 3T Research Centre, IRCCS Mondino Foundation, 27100 Pavia, Italy; NMR Research Unit, Queen Square Multiple Sclerosis Centre, University College London Queen Square Institute of Neurology, London WC1N 3BG, UK; NMR Research Unit, Queen Square Multiple Sclerosis Centre, University College London Queen Square Institute of Neurology, London WC1N 3BG, UK; National Institute for Health and Care Research (NIHR) University College London Hospitals (UCLH) Biomedical Research Centre, London W1T 7DN, UK; NMR Research Unit, Queen Square Multiple Sclerosis Centre, University College London Queen Square Institute of Neurology, London WC1N 3BG, UK; National Institute for Health and Care Research (NIHR) University College London Hospitals (UCLH) Biomedical Research Centre, London W1T 7DN, UK; NMR Research Unit, Queen Square Multiple Sclerosis Centre, University College London Queen Square Institute of Neurology, London WC1N 3BG, UK; Centre for Medical Image Computing (CMIC), Department of Medical Physics and Biomedical Engineering, University College London, London WC1E 6BT, UK; National Institute for Health and Care Research (NIHR) University College London Hospitals (UCLH) Biomedical Research Centre, London W1T 7DN, UK; Department of Radiology and Nuclear Medicine, VU University Medical Centre, 1081 HV Amsterdam, The Netherlands; NMR Research Unit, Queen Square Multiple Sclerosis Centre, University College London Queen Square Institute of Neurology, London WC1N 3BG, UK; MS Centre of Catalonia (Cemcat), Vall d'Hebron Institute of Research, Vall d'Hebron Barcelona Hospital Campus, 08035 Barcelona, Spain; Department of Neuromuscular Diseases, UCL Queen Square Institute of Neurology, Queen’s Square House, Queen’s Square, London, WC1N 3BG, UK; NMR Research Unit, Queen Square Multiple Sclerosis Centre, University College London Queen Square Institute of Neurology, London WC1N 3BG, UK; National Institute for Health and Care Research (NIHR) University College London Hospitals (UCLH) Biomedical Research Centre, London W1T 7DN, UK

**Keywords:** multiple sclerosis, disease progression, severity, genetics, phenotype

## Abstract

Multiple sclerosis risk has a well-established polygenic component, yet the genetic contribution to disease course and severity remains unclear and difficult to examine. Accurately measuring disease progression requires long-term study of clinical and radiological outcomes with sufficient follow-up duration to confidently confirm disability accrual and multiple sclerosis phenotypes. In this retrospective study, we explore genetic influences on long-term disease course and severity; in a unique cohort of clinically isolated syndrome patients with homogenous 30-year disease duration, deep clinical phenotyping and advanced MRI metrics. Sixty-one clinically isolated syndrome patients [41 female (67%): 20 male (33%)] underwent clinical and MRI assessment at baseline, 1-, 5-, 10-, 14-, 20- and 30-year follow-up (mean age ± standard deviation: 60.9 ± 6.5 years). After 30 years, 29 patients developed relapsing-remitting multiple sclerosis, 15 developed secondary progressive multiple sclerosis and 17 still had a clinically isolated syndrome. Twenty-seven genes were investigated for associations with clinical outcomes [including disease course and Expanded Disability Status Scale (EDSS)] and brain MRI (including white matter lesions, cortical lesions, and brain tissue volumes) at the 30-year follow-up. Genetic associations with changes in EDSS, relapses, white matter lesions and brain atrophy (third ventricular and medullary measurements) over 30 years were assessed using mixed-effects models. HLA-DRB1*1501-positive (*n* = 26) patients showed faster white matter lesion accrual [+1.96 lesions/year (0.64–3.29), *P* = 3.8 × 10^−3^], greater 30-year white matter lesion volumes [+11.60 ml, (5.49–18.29), *P* = 1.27 × 10^−3^] and higher annualized relapse rates [+0.06 relapses/year (0.005–0.11), *P* = 0.031] compared with HLA-DRB1*1501-negative patients (*n* = 35). *PVRL2*-positive patients (*n* = 41) had more cortical lesions (+0.83 [0.08–1.66], *P* = 0.042), faster EDSS worsening [+0.06 points/year (0.02–0.11), *P* = 0.010], greater 30-year EDSS [+1.72 (0.49–2.93), *P* = 0.013; multiple sclerosis cases: +2.60 (1.30–3.87), *P* = 2.02 × 10^−3^], and greater risk of secondary progressive multiple sclerosis [odds ratio (OR) = 12.25 (1.15–23.10), *P* = 0.031] than *PVRL2*-negative patients (*n* = 18). In contrast, *IRX1*-positive (*n* = 30) patients had preserved 30-year grey matter fraction [+0.76% (0.28–1.29), *P* = 8.4 × 10^−3^], lower risk of cortical lesions [OR = 0.22 (0.05–0.99), *P* = 0.049] and lower 30-year EDSS [−1.35 (−0.87,−3.44), *P* = 0.026; multiple sclerosis cases: −2.12 (−0.87, −3.44), *P* = 5.02 × 10^−3^] than *IRX1*-negative patients (*n* = 30). In multiple sclerosis cases, *IRX1*-positive patients also had slower EDSS worsening [−0.07 points/year (−0.01,−0.13), *P* = 0.015] and lower risk of secondary progressive multiple sclerosis [OR = 0.19 (0.04–0.92), *P* = 0.042]. These exploratory findings support diverse genetic influences on pathological mechanisms associated with multiple sclerosis disease course. HLA-DRB1*1501 influenced white matter inflammation and relapses, while *IRX1* (protective) and *PVRL2* (adverse) were associated with grey matter pathology (cortical lesions and atrophy), long-term disability worsening and the risk of developing secondary progressive multiple sclerosis.

## Teaser

Sahi *et al*. report genetic associations with clinical and MRI outcomes 30 years after a clinically isolated syndrome suggestive of multiple sclerosis. HLA-DRB1*1501 is associated with greater white matter inflammation and relapses, while *IRX1* (protective) and *PVRL2* (adverse) influenced grey matter pathology, long-term disability and risk of secondary progressive multiple sclerosis.

## Graphical Abstract

**Graphical abstract:**
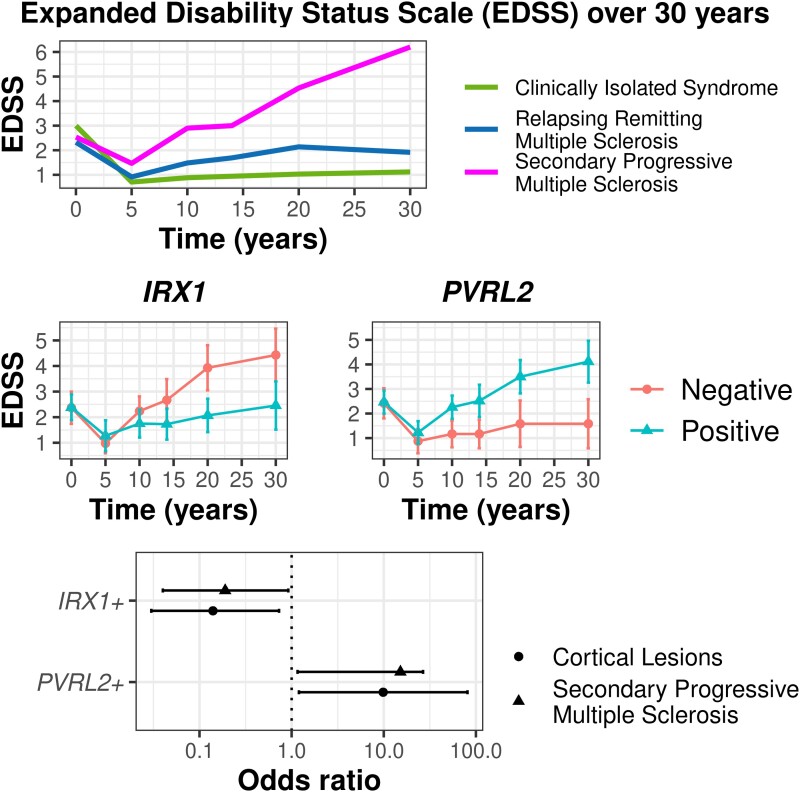

## Introduction

Multiple sclerosis (MS) is a clinically and radiologically diverse condition. Some people with MS accrue little disability and show few radiological signs over many years, while others develop marked neurological deficits and MRI changes. We do not know why MS is so heterogeneous, but genetic factors are likely to be relevant.

Genome-wide association studies (GWAS) have identified over 200 MS-susceptibility variants^1^ but establishing genetic contribution to MS severity has proven more difficult. Although studies have suggested potential genotype-phenotype associations (see Ref. 2 for a review), the genetic contribution to disease severity or clinical course proved elusive in early GWAS.^3–5^ Recently, the International Multiple Sclerosis Genetics Consortium conducted a GWAS that has identified a variant (rs10191329) associated with age-related MS severity.^6^ A longitudinal GWAS has also identified two single nucleotide polymorphisms (SNPs) (rs10967273 in females and rs698805 in males) associated with Multiple Sclerosis Severity Score (MSSS) in sex-stratified analyses.^7^ Both studies required large-scale multi-centre collaboration and included participants with longer disease duration and follow-up (mean disease duration 18.2 years in Ref. 6 and median disease duration 18.1 years in Ref. 7). Even within longitudinal studies, variability and limited duration of follow-up^7^ may weaken or obscure associations with genetic factors, as nearly half of people with MS do not develop clinically significant disability in the first decade and severe disability or progression to secondary progressive multiple sclerosis (SPMS) may not occur for 20 years or more.^8^ Genotype-phenotype correlation in people who had had a clinically isolated syndrome (CIS) with homogeneous 15-year disease duration showed HLA-DRB1*1501 status influenced inflammatory disease activity, brain atrophy and long-term disability, but was limited to an assessment of only one gene.^9^

We recently completed a 30-year prospective follow-up study of a CIS cohort.^10^ In those with known outcomes, a third remained classified as CIS, a third developed SPMS or MS-related death, and the rest had RRMS, nearly all remaining fully ambulatory. Leading up to the 30-year follow-up there were significant differences in the trajectories of lesion accrual, brain atrophy and disability progression in these groups,^10,11^ and at 30 years cortical abnormalities, in particular cortical lesions, were the most distinguishing feature between RRMS and SPMS.^12^ For some the path to SPMS was apparent very early on, with the presence of infratentorial and deep white matter (WM) lesions within 1 year as significant predictors of developing SPMS.^10^

In this exploratory study, we utilize the strengths of this unique cohort with homogenous 30-year disease duration, deep clinical phenotyping and advanced MRI metrics to explore potential genetic associations with clinical progression and underlying pathological mechanisms. We aimed to replicate previously seen associations, whilst also hypothesizing that genes associated with inflammation would influence relapse activity and genes linked with neurodegeneration would influence progressive disability and disease phenotype.

## Materials and methods

### Study cohort

This cohort has previously been described.^10^ Briefly, 132 people with a CIS were recruited prospectively (1984–1987) at the National Hospital of Neurology and Neurosurgery, and Moorfields Eye Hospital, and followed over 30 years. Participants underwent clinical assessment and MRI brain at baseline, 1, 5, 10, 14, 20 and 30 years. Previous reports have described MRI and clinical outcomes.^10–12^ At 30-year follow-up, 61 participants consented and gave blood samples for genotyping. Participants were classified with either CIS (17/61) or MS (44/61) using 2010 McDonald diagnostic criteria,^13^ and 15 had progressed to SPMS using Lublin criteria^14^ (*Table 1*). These patients were included in this study, of which only 9 patients received disease modifying therapy (DMT), all with first-line injectable drugs and earliest DMT use was 10 years after MS diagnosis.

**Table 1:** Demographics with clinical and radiological characteristics by 30-year clinical diagnosis

		Diagnosis at 30 years		
	All participants	CIS	RRMS	SPMS
Number	61	17	29	15
Age (years)	60.9 ± 6.5	60.6 ± 6.8	60.6 ± 6.4	61.9 ± 6.7
Female	41 (67%)	10 (59%)	20 (69%)	11 (73%)
HLA-DRB1*1501+	26 (43%)	5 (29%)	16 (55%)	7 (47%)
Age at Onset (years)	30.2 ± 6.4	30.2 ± 7.3	29.6 ± 6.3	31.6 ± 6.2
Disease Duration (years)	30.8 ± 0.9	30.6 ± 0.9	30.8 ± 1.0	30.8 ± 0.9
CIS Type				
*Optic Neuritis*	31 (51%)	10 (59%)	13 (45%)	8 (53%)
*Spinal cord*	21 (34%)	6 (35%)	10 (35%)	5 (33%)
*Brainstem*	9 (15%)	1 (6%)	6 (21%)	2 (13%)
Baseline EDSS^a^				
*Mean ± SD*	2.6 ± 1.3	3.0 ± 1.2	2.3 ± 1.0	2.5 ± 1.7
*Median (IQR)*	3.0 (2.0–3.125)	3.0 (3.0–3.375)	2.0 (2.0–3.0)	3.0 (1.0–3.5)
OCBs				
*Positive*	25	2	13	10
*Negative*	6	5	1	0
*Unknown*	20	10	15	5
Time CIS to RRMS (years)	5.8 ± 6.0	NA	6.7 ± 6.9	3.8 ± 3.9
Time CIS to SPMS (years)	19.6 ± 5.5	NA	NA	19.6 ± 5.5
EDSS at 30 years				
*Mean ± SD*	2.7 ± 2.4	1.1 ± 1.1	1.9 ± 1.6	6.2 ± 0.8
*Median (IQR)*	2.0 (1–5.5)	1.0 (0.0–2.0)	1.5 (1.0–2.25)	6.0 (6.0–6.5)
DMT usage				
*Yes*	9	0	4	5
*No*	52	17	25	10
Smoking history (at 30 years)				
*Current*	8	3	3	2
*Ex*	29	7	14	8
*Never*	24	7	12	5
Baseline WM lesion counts^b^	8.0 ± 15.8	0.5 ± 1.1	10.0 ± 15.9	12.0 ± 21.3
Baseline WM lesion volume (ml)	1.17 ± 2.37	0.08 ± 0.017	0.85 ± 0.77	2.46 ± 3.94

CIS, clinically isolated syndrome; RRMS, relapsing-remitting multiple sclerosis; SPMS, secondary progressive multiple sclerosis; EDSS, expanded disease severity scale; IQR, interquartile range, OCBs, oligoclonal bands; DMT, disease modifying therapy; WM, white matter.

Mean ± standard deviations unless stated otherwise.

^a^Baseline EDSS was recorded during initial CIS presentation.

^b^WM lesions were marked by consensus of at least two-trained assessors blinded to clinical status, and counted manually by K.C as per Chung *et al*.^10^

This study was approved by the National Research Ethics Service (15/LO/0650). All participants gave written informed consent.

### Clinical assessment

Disability was assessed at baseline, 5-, 10-, 14-, 20- and 30-year follow-ups, using EDSS by examination (or telephone at later timepoints) and determined retrospectively if missing at a given follow-up.^10^ Relapse history was obtained at 5-, 10-, 20- and 30-year follow-up. At 30-year follow-up, additional assessments included the timed 25-foot Walk Test (T25FWT), 9-Hole Peg Test (9HPT) and neuropsychological assessments; Paced Auditory Serial Addition Test 3 (PASAT-3), Fatigue Severity Scale (FSS) and Brief International Cognitive Assessment for Multiple Sclerosis (BICAMS), comprising 3 components [Brief Visuospatial Memory Test–Revised (BVMTR), Symbol Digit Modalities Test (SDMT) and California Verbal Learning Test (CVLT)] with z-scores adjusted for age, sex and years of education.^10^

### MRI acquisition

Baseline, 1-year and 5-year MRI scans were obtained on a 0.5T scanner (Picker, Cleveland), a 1.5T scanner (General Electric Signa Echospeed, Milwaukee) at 10, 14 and 20 years, and a 3T Philips Achieva at 30 years (see Ref. 11) for full acquisition parameters. Proton density and/or T2-weighted scans were obtained at each timepoint. Acquisition parameters of the 30-year MRI images and analysis are detailed previously^12^ and included 3D fluid-attenuated inversion recovery, T1-weighted volumetric images, magnetization transfer ratio (MTR) and phase-sensitive inversion recovery (PSIR).

### MRI analysis

MRI analysis methods have been detailed previously.^10–12^ Briefly, WM lesion counting was performed manually and blinded to clinical status for all timepoints.^10^ T2-weighted lesion volume measures were available for baseline, 5, 10, 14, 20 and 30 years but were not available for the 1-year follow-up.^15^ Third ventricular width (TVW) and medullary width (MEDW) were obtained as linear measures of brain atrophy at all follow-ups.^11^

At 30-year follow-up, cortical lesions were counted manually on PSIR and grey matter fraction (GMF) was derived from T1-weighted volumetric images using Geodesic Information Flows.^16^ MTR maps [in percentage units (pu)] were calculated for normal-appearing WM, WM lesions, cortical grey matter (GM) and deep GM.^12^

The clinical and radiological outcome measures at each follow-up used for cross-sectional and longitudinal analysis are shown (*Table 2*).

**Table 2:** Quantified radiological and clinical outcome measures analysed at each follow-up timepoint

Datapoints, *n*		Follow-up (years)							
Outcome		0	1	5	10	14	20	30	Total^a^
**Inflammation**									
WM lesion count *(n)*		60	48	49	41	36	52	59	**345**
WM lesion volume *(ml)*		39	-	41	40	35	49	59	**263**
WM lesion MTR *(pu)*		-	-	-	-	-	-	55	
Normal appearing WM MTR *(pu)*		-	-	-	-	-	-	55	
**Brain volume**									
GM fraction *(%)*		-	-	-	-	-	-	59	
TVW *(mm)*		53	46	48	41	36	53	58	**335**
MEDW *(mm)*		35	30	46	37	33	51	57	**289**
**Clinical**									
**Neuropsychology**									
BVMTR-z		-	-	-	-	-	-	44	
BICAMS		-	-	-	-	-	-	55	
CVLT-z		-	-	-	-	-	-	45	
FSS		-	-	-	-	-	-	56	
PASAT-3		-	-	-	-	-	-	57	
SDMT-z		-	-	-	-	-	-	44	
**Severity / Disability**									
Relapses *(n)*		-	-	61	58	-	59	61	**239**
EDSS		58	-	61	61	61	61	61	**363**
9HPT-D *(s)*		-	-	-	-	-	-	61	
9HPT-ND *(s)*		-	-	-	-	-	-	61	
T25FTW *(s)*		-	-	-	-	-	-	58	
**Phenotype**									
Diagnosis		-	-	-	-	-	-	61	

Table of quantified radiological and clinical outcomes analysed at 30 years (cross-sectional) and over 30 years (longitudinal). Some outcome measures did have data available at other time points but were not available from study onset and hence were not included in longitudinal analyses.

WML, white matter; MTR, magnetization transfer ratio; GM, grey matter; TVW, third ventricular width; MEDW, medullary width; BVMTR-z—Brief Visuospatial Memory Test–Revised z-score adjusted for age; sex and education; BICAMS, brief international cognitive assessment for MS; CVLT-z, California verbal learning test z-score; FSS, fatigue severity scale; PASAT-3, paced serial auditory test-3; SDMT-z, Symbol digit Modalities Test z-score; 9HPT-D, 9-hole peg test dominant hand; 9HPT-ND, 9-hole peg test non-dominant hand; T25FTW, timed 25 ft walk; EDSS -expanded disability status scale; *pu*, percentage units.

^a^Total datapoints available for longitudinal analysis are indicated in bold, *n* Number of study participants = 61.

### SNP selection and genotyping

Literature review identified 38 genetic variants putatively associated with inflammation, neurodegeneration, or clinical features in MS, with tagging SNPs identifiable using the Infinium Global Screening Array-24 v3.0 Beadchip from Illumina (San Diego, CA, USA) (*see*Supplementary Table 1*for SNP selection data*).

DNA was genotyped by the University College London Genomics Laboratory. Genotyped data was assembled in GenomeStudio v2.0.5 (Illumina). Quality control excluded eleven SNPs with minor allele frequency <0.05, call rate <95%, deviation from Hardy-Weinberg equilibrium (*P* < 0.05) or individual heterozygosity rate >3 standard deviations from mean,^17^ leaving 27 SNPs for analysis.

### Statistical analysis

#### 30-year cross-sectional associations

The association between each SNP and phenotype at 30 years was assessed using logistic regression with ‘diagnosis of SPMS (‘yes’ or ‘no’) at 30 years’ as the dependent variable with binary SNP (‘positive’ or ‘negative’ assuming a dominant genetic model) as the independent variable. Linear regression models were built with continuous clinical or radiological variables (one at a time) at baseline and 30-year follow-up as the dependent variable, SNP the main predictor. All models included age, sex, DMT use (‘yes’ or ‘no’) and smoking history (‘current’, ‘ex-smoker’, or ‘never’) as covariates. Linearity assumptions of continuous predictors (Age) with their logit were tested using the Box-Tidwell test.

#### Longitudinal associations (0–30 years)

Associations between each SNP and changes in clinical (relapse count and EDSS) and MRI variables (WM lesion count, WM lesion volume, TVW and MEDW) over the 30-year follow-up period were assessed using generalized linear mixed-effects models. These models specified ‘annualized change in outcome variable’ between each timepoint as the dependent variable (e.g. annualized change between 0–5 years at 5-year follow-up, between 5–10 years at 10-year follow-up) with SNP as the main predictor and age, sex, DMT use and smoking history included as covariates. The models specified a normal distribution with identity link, random intercept and random slope. The Akaike information criterion was used in model selection and to assess goodness of fit of models to the data. Whenever the SNP was significant (*P* < 0.05), we understood that it influenced the annualized change in the dependent variable at each timepoint.

For all models, testing both cross-sectional and longitudinal associations, subgroup analysis was conducted only on those who developed MS, excluding participants who remained as CIS after 30 years. This was to ensure any genetic associations that influenced outcomes within MS patients were not due to over-representation of genotypes within CIS participants.

For all linear models, we checked model assumptions, including normality of residuals and homoscedasticity. If assumptions were not met, bootstrap-based methods were used to estimate 95% confidence intervals.

In this study, we report all results with *P*-value <0.05. However, we only interpreted associations between the different SNPs and the clinical and radiological variables as being plausible when we observed at least two biologically consistent results for a given SNP. Isolated associations or biologically contradicting observations for a given SNP were taken with caution and interpreted as inconclusive.

We used SPSS (Version 27, Chicago, IL, USA) for statistical analysis and RStudio (Version 1.1.463, Boston, MA, USA) for data visualization.

## Results

### Baseline characteristics and associations

Participant demographics are shown by 30-year MS phenotype. (*Table 1*) *DKK2* was associated with more baseline lesions [+9.7 lesions, bootstrapped 95% CI: (−1.2, 24.1), *P* = 0.047]. *HDAC9* was associated with lower baseline EDSS [−0.89, bootstrapped 95% CI: (−0.31, −1.57), *P* = 0.011] and *LRRC41* with lower baseline TVW [−0.62 mm, 95% CI: (−0.04, −1.19), *P* = 0.037]. No other SNPs showed baseline differences in WM lesion measures, EDSS, MEDW or TVW between genetic groups.

### Cross-sectional genetic associations with 30-year outcomes

Five SNPs (HLA-DRB1*1501, *BATF, PVRL2, IRX1* and *HDAC9*) showed biologically plausible associations with clinical and radiological outcome measures at 30 years. (*Table 3*) All individual 30-year cross-sectional genetic associations are summarized in Supplementary*Tables 2 and 3*.

**Table 3:** SNPs with multiple *p* < 0.05 associations with MRI and clinical measures at 30-year follow-up

			HLA-DRB1*1501	*BATF*	*PVRL2*	*IRX1*	*HDAC9*
**Inflammation** White Matter	Lesion count *(n)* Lesion Volume *(ml)*	Est 95% CI *P*-value Est 95% CI *P*-value	54.99 3.60, 104.79^a^ 0.021 11.60 5.49, 18.29^a^ 1.27 × 10^−3^**				
	Lesional MTR *(pu)* NAWM MTR *(pu)*	Est 95% CI *P*-value Est 95% CI R^2^ *P*-value	−1.17 −0.24, −2.26^a^ 0.013 −0.85 −0.14, −1.66^a^ 0.20 0.021				
Grey Matter	GM lesions (*n)*	Est 95% CI *P*-value		−0.81 −0.20, −1.41^a^ 0.029	0.83 0.08, 1.66^a^ 0.042		
	GM lesion *(OR)*	Est 95% CI *P*-value		0.09 0.01, 0.81 0.032	7.23 1.13, 46.21 0.037	0.22 0.05, 0.99 0.049	
	cGM MTR gradient (pu/band)	Est 95% CI *P*-value				0.15 0.02, 0.29^a^ 0.019	
**Brain Volume**	GMF (%)	Est 95% CI *P*-value	−0.62 0.00, −1.36^a^ 0.030			0.76 0.28, 1.29^a^ 8.40 × 10^−3^*	−0.72 −0.10, −1.37^a^ 0.014
	MEDW *(mm)*	Est 95% CI *P*-value				0.28 0.03, 0.46^a^ 0.042	
	TVW *(mm)*	Est 95% CI *P*-value				−1.82 −0.52, −3.22^a^ 0.014	
**Clinical** Cognitive Fatigue	SDMT-z	Est 95% CI *P*-value		0.715 0.02, 1.42 0.046			0.736 0.05, 1.42 0.035
	BICAMS Total	Est 95% CI *P*-value		15.23 4.94, 26.31^a^ 0.016			
	FSS	Est 95% CI *P*-value				−7.98 −0.10,−15.87^a^ 0.047	12.92 3.05, 18.81 7.58 × 10^−3^*
Disability	9HPT-ND *(s)*	Est 95% CI *P*-value				−6.38 −2.32, −11.14 9.35 × 10^−3^**	
	EDSS	Est 95% CI *P*-value			1.72 0.49, 2.93^a^ 0.013	−1.35 −0.29, −2.48^a^ 0.026	
Phenotype	Progression to SPMS *(OR)*	Est 95% CI *P*-value			12.25 1.15, 23.10^a^ 0.031		

NAWM, normal appearing white matter; GM, grey matter; cGM, cortical grey matter; MTR, magnetization transfer ratio; pu, percentage units; GMF, grey matter fraction; TVW, third ventricular width; MEDW, medullary width; SDMT-z, Symbol digit Modalities Test z-score adjusted for age; sex and education; BICAMS, brief international cognitive assessment for MS; FSS, fatigue severity scale; 9HPT-ND, 9-hole peg test non-dominant hand; EDSS -expanded disability status scale; SPMS, Secondary progressive MS. All Estimated co-efficients from linear regression models and odds ratios (OR) from logistic regression models included age; sex, DMT use and smoking history as covariates.

^a^Bootstrapped confidence intervals using 1000 replicates.

The *P*-values of the most statistically significant associations observed are highlighted in bold.

^*^ *P* *< 0.01*.

^**^ *P <* 1.85 × 10^−3^*(*Bonferroni correction for number of variants = 0.05/27 variants).

#### White matter MRI measures

HLA-DRB1*1501 carriage was associated with a greater WM lesion volume [+11.60 ml, bootstrapped 95% CI: (5.49, 18.29), *P* = 1.27 × 10^−3^], number [+54.99 lesions, bootstrapped 95% CI: (3.60, 104.79), *P* = 0.021], lower MTR within lesions [−1.17pu, bootstrapped 95% CI: (−0.24, −2.26), *P* = 0.013] and within normal-appearing WM [−0.85pu, bootstrapped 95% CI: (−0.14, −1.66), *P* = 0.021]. No other SNPs showed associations with WM inflammation or MTR measures.

#### Grey matter MRI measures

*BATF* and *IRX1* reduced the odds of cortical lesions [*BATF*: OR 0.09, 95% CI: (0.01, 0.81), *P* = 0.032 and *IRX1*: OR 0.22, 95% CI (0.05, 0.99) *P* = 0.049] and *PVRL2* increased the odds [OR 7.23, 95% CI: (1.13, 46.21), *P* = 0.037]. *PVRL2* correlated with more cortical lesions [+0.83, bootstrapped 95% CI: (0.08, 1.66), *P* = 0.042] and *BATF* with fewer cortical lesions [−0.81, bootstrapped 95% CI: (−0.20, −1.41), *P* = 0.029]. *IRX1* was associated with shallower (less negative) cortical GM MTR gradients [+0.15 pu/band, bootstrapped 95% CI: (0.02, 0.29), *P* = 0.019] in the whole cohort but this was not replicated in MS-only analysis.

#### Brain volume

*IRX1* was associated with higher GMF [+0.76%, bootstrapped 95% CI: (0.28,1.29), *P* = 8.4 × 10^−3^] and linear brain measures, with greater MEDW [+0.28 mm, 95% CI: (0.03, 0.46), *P* = 0.042] and lower TVW [−1.82 mm, bootstrapped 95% CI: (−0.52, −3.22), *P* = 0.014].

*HDAC9* [−0.72%, bootstrapped 95% CI: (−0.10, −1.37), *P* = 0.014] and HLA-DRB1*1501 correlated with reduced 30-year GMF [−0.62%, bootstrapped 95% CI: (0.00, −1.36), *P* = 0.030], although HLA-DRB1*1501 was not significant after including WM lesion volume within the model (HLA-DRB1*1501; P = 0.239; WM lesion volume *P* = 0.039) and no association was seen between HLA-DRB1*1501 and GMF in the MS-only subgroup.

#### Cognition and fatigue

Whole group associations of *BATF,* with better SDMT-z scores [+0.715, 95% CI: (0.02, 1.42), *P* = 0.046] and overall BICAMS scores [+15.23, bootstrapped 95% CI: (4.94, 26.31), *P* = 0.016], and *HDAC9,* with better SDMT-z scores [+0.736, 95% CI: (0.05, 1.42), *P* = 0.035], were not seen in MS patients and appeared driven by differences in those with CIS.

HDAC9 was associated with greater fatigue on FSS [+12.92, bootstrapped 95% CI: (3.05,18.81), *P* = 7.58 × 10^−3^] while *IRX1* was associated with lower fatigue scores [−7.98, bootstrapped 95% CI: (−0.10, −15,87), *P* = 0.047].

#### Physical disability and disease phenotype

**Figure 1:**
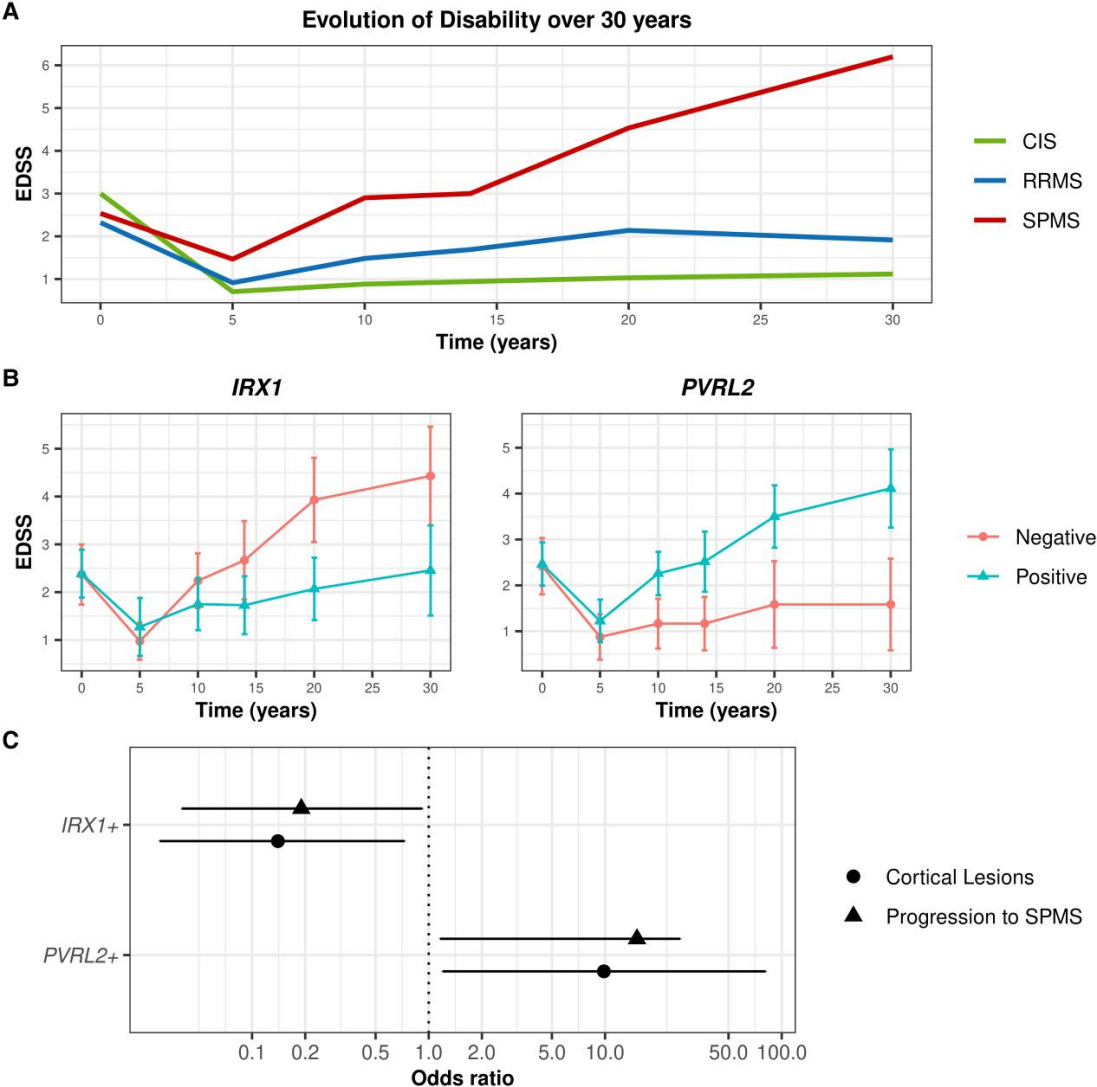
**Associations of *IRX1* and *PVRL2* with disability, cortical GM lesions and SPMS phenotype in participants with MS.** CIS—clinically isolated syndrome, RRMS—relapsing-remitting multiple sclerosis, SPMS—secondary progressive multiple sclerosis, EDSS—expanded disability status scale. (**A**) Observed mean EDSS over time by 30-year clinical phenotype in the whole cohort (*n* = 61). (**B**) Observed mean EDSS over time with 95% CIs at each timepoint by *IRX1* and *PVRL2* in those with MS (*n* = 43). Associations between each SNP and annualized change in EDSS over the 30-year follow-up period were assessed using linear mixed-effects models including age, sex, DMT use, and smoking history as covariates. *PVRL2* influenced annualized EDSS accrual [+0.09 points/year, 95% CI: (0.02, 0.015), *P* = 0.012] while *IRX1* was also associated with slower annualized EDSS changes by −0.07 points/year [95% CI: (−0.13, −0.01), *P* = 0.015]. (**C**) Forest plots showing the estimated odds ratio from logistic regression models assessing the presence of GM lesions and progression to SPMS phenotypes for *IRX1* and *PVRL2* genotypes as a binary outcome (Yes/No) adjusted for age, gender, disease-modifying therapy use and smoking history. In MS patients (*n* = 43), *IRX1* was associated with reduced risk of cortical lesions [OR 0.14, 95% CI (0.03, 0.73) *P* = 0.020] and *PVRL2* with increased risk of cortical lesions [OR 9.85, 95% CI: (1.20, 81.04), *P* = 0.037]. Both genes influenced risk of progression to SPMS [*PVRL2:* OR 15.16, bootstrapped 95% CI: (1.16, 26.67), *P* = 0.031; *IRX1:* OR 0.19, 95% CI: (0.04, 0.92), *P* = 0.042].

*PVRL2* was associated with 1.72-point higher 30-year EDSS [bootstrapped 95% CI: (0.49, 2.93), *P* = 0.013] and *IRX1* with 1.35-point lower EDSS [bootstrapped 95% CI: (−0.29, 2.48), *P* = 0.026]. *IRX1* also correlated with better 9HPT-ND performance [−6.38 seconds, 95% CI: (−2.32, −11.14), *P* = 9.35 × 10^−3^]. *PVRL2* positivity was associated with increased risk of SPMS [OR 12.25, bootstrapped 95% CI: (1.15, 23.10), *P* = 0.031] (*Figure 1*) with 93.3% sensitivity (14/15 SPMS patients), and 94.4% negative predictive value (17/18 negative genetic tests). No significant association was found between HLA-DRB1*1501 and disability or disease phenotype.

In MS participants only, *PVRL2* and *IRX1* had clearer associations with 30-year EDSS [*PVRL2: + 2.60*, bootstrapped 95% CI: (1.30, 3.87), *P* = 2.02 × 10^−3^; *IRX1: −2.12,* bootstrapped 95% CI: (−0.87, −3.44), *P* = 5.02 × 10^−3^] and both influenced risk of SPMS [*PVRL2:* OR 15.16, bootstrapped 95% CI: (1.16, 26.67), *P* = 0.031; *IRX1:* OR 0.19, 95% CI: (0.04, 0.92), *P* = 0.042).

### Genetic associations with longitudinal outcome measures

In the whole (CIS and MS) cohort, seven genes (HLA-DRB1*1501, *AHI1, KCNIP1, IRX1, HDAC9, PVRL2* and *EVI5*) showed associations with outcomes measures assessed from 0 to 30 years using longitudinal mixed-effects models. (*Table 4*) Subgroup analysis of those who developed MS showed associations of HLA-DRB1*1501, AHI1, IRX1 and *PVRL2* with longitudinal changes in outcome measures over 30 years. (*Table 5*)

**Table 4:** SNP associations with longitudinal (0–30 years) radiological and clinical measures (*p* < 0.05)

Annualized change between each follow-up timepoint		Gene, *SNP (allele)*						
		HLA- DRB1*1501 *rs3135388 (T)*	AHI1 *rs11154801 (T)*	KCNIP1 *rs11957313* *(A)*	IRX1 *rs4866550* *(T)*	HDAC9 *rs2074633* *(C)*	PVRL2 *rs4803766* *(A)*	EVI5 *rs11810217* *(A)*
**Inflammation**								
WM lesions *(n / yr)*	Estimate 95% CI *P*-value	1.96 0.64, 3.29 3.8 × 10^−3^*						
WM lesion volume *(ml / yr)*	Estimate 95% CI	0.29 0.06, 0.53						
	*P*-value	0.015						
**Brain Volume**								
TVW *(mm / yr)*	Estimate 95% CI *P*-value			0.05 0.01, 0.10 0.022	−0.06 0.01, 0.10 0.016			
**Clinical**								
Relapses (*n* / yr)	Estimate 95% CI *P*-value		0.04 0.01, 0.08 0.047					
EDSS (points / yr)	Estimate 95% CI *P*-value					0.05 0.01, 0.10	0.06 0.02, 0.11	0.05 0.01, 0.10
						0.03	**0.01** *	0.03

All significant (*P < 0.05)* associations from generalized linear mixed-effects models adjusting for age, sex, DMT use, and smoking history assessed either effect of SNP genotype (positive versus negative) on annualized rate of change of outcome measure between timepoints.

CI, confidence interval; SNP, single nucleotide polymorphism; WM, white matter; TVW, third ventricular width; EDSS, expanded disability status scale.

The *P*-values of the most statistically significant associations observed are highlighted in bold.

^*^ *P < 0.01*.

Bonferroni correction for number of variants = 0.05/27 variants = *P < 1.85 × 10^−3^*.

**Table 5:** Longitudinal SNP associations (0–30 years) with radiological and clinical measures (*p* < 0.05) in MS participants only (excluding CIS)

Annualized change between each follow-up timepoint		Gene, *SNP (Allele)*			
		HLA- DRB1*1501 rs3135388 (T)	*AHI1* rs11154801 (T)	*IRX1* rs4866550 (T)	*PVRL2* rs4803766 (A)
**Inflammation**					
WM lesions (*n / yr)*	Estimate 95% CI *P*-value	2.23 0.72, 3.73 3.9 × 10^−3^*	1.94 0.21, 3.67 0.029		
WM lesion volume *(ml / yr)*	Estimate 95% CI *P*-value	0.30 0.02, 0.58 0.035			
**Clinical**					
Relapses (*n / yr)*	Estimate 95% CI *P*-value	0.06 0.005, 0.11 0.031	0.06 0.002, 0.11 0.041		
EDSS (points / yr)	Estimate 95% CI *P*-value			−0.07 −0.01, −0.13	0.09 0.02, 0.15
				0.015	0.012

All significant (*P <* 0.05) associations from generalized linear mixed-effects models adjusting for age, sex, DMT use, and smoking history assessed either effect of SNP genotype (positive versus negative) on annualized rate of change of outcome measure between timepoints.

CI, confidence interval; SNP, single nucleotide polymorphism; WM, white matter lesion; EDSS, expanded disability status scale.

The *P*-values of most statistically significant associations observed are highlighted in bold.

^*^ *P* < 0.01.

Bonferroni correction for number of variants = 0.05/27 variants = *P <* 1.85 × 10^−3^.

#### Changes in white matter MRI measures

**Figure 2:**
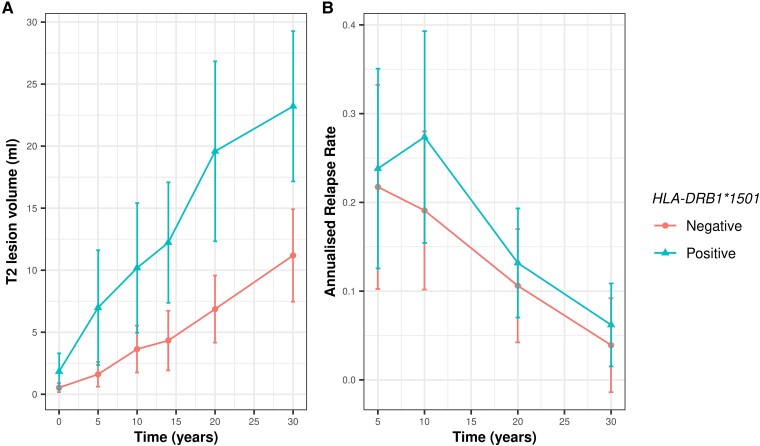
Associations of HLA-DRB1*1501 with white matter lesion volume and annualized relapse rates in participants with MS. HLA-DRB1*1501 associations between annualized change in white matter T2 lesion volume and annualized relapse rate over the 30-year follow-up period were assessed using linear mixed-effects models including age, sex, disease modifying therapy use, and smoking history as covariates. (*A*) Observed mean white matter T2 lesion volume (ml) over time by HLA-DRB1*1501 genotype in the whole cohort (*n* = 61). HLA-DRB1*1501 was associated with greater annualized change in white matter lesion volume by 0.29 ml/year [95% CI: (0.06–0.53), *P* = 0.0150 in the whole cohort and in MS-only subgroup analysis [*n* = 44 ,+ 0.30 ml/year, 95% CI: (0.02, 0.58), *P* = 0.035]. (**B**) Observed mean annualized relapse rate over time by HLA-DRB1*1501 gene expression in MS patients only (*n* = 44). HLA-DRB1*1501 was associated with higher annualized relapse rates [+0.06 relapses/year, 95% CI: (0.005, 0.11), *P* = 0.031]. Error bars represent 95% confidence intervals.

HLA-DRB1*1501 was associated with greater annualized change in WM lesion number [+1.96 lesions/year, 95% CI: (0.64–3.29), *P* = 3.8 × 10^−3^] and with greater annualized change in WM lesion volume by 0.29 ml/year [95% CI: (0.06–0.53), *P* = 0.015] in the whole cohort (*Figure 2*). In those who developed MS, HLA-DRB1*1501 remained associated with faster increase in WM lesion number [+2.23 lesions/year, 95% CI: (0.72, 3.73), *P* = 3.9 × 10^−3^] and greater annualized change in WM lesion volume [+0.30 ml/year, 95% CI: (0.02, 0.58), *P* = 0.035]. *AHI* was also associated with faster WM lesion accrual [+1.94 lesions/year, 95% CI: (0.21, 3.67), *P* = 0.029] in MS patients.

#### Changes in brain volume

*IRX1* was associated with slower increase in TVW over time [−0.06 mm/year, 95% CI: (0.01–0.10), *P* = 0.016] and *KCNIP1* with faster increase in TVW (+0.05 mm/year, 95% CI: 0.01–0.10, *P* = 0.022) in whole group analysis but neither were replicated in the MS-only subgroup.

#### Relapses

Overall, the 44 people with MS had 166 relapses over 30 years following initial CIS event. *AHI1* was associated with higher annualized relapse rates in the whole CIS and MS cohort (+0.04 relapses/year, 95%CI: 0.01–0.08, *P* = 0.047) and in MS patients only (+0.06 relapses/year, 95%CI: 0.002–0.11, *P* = 0.041). In MS patients, HLA-DRB1*1501 was associated with higher annualized relapse rates [+0.06 relapses/year, 95% CI: (0.005, 0.11), *P* = 0.031].

#### Changes in physical disability and disease phenotype

*PVRL2* influenced annualized EDSS accrual (+0.06 points/year, 95%CI: 0.02–0.11, *P* = 0.010) in the whole cohort and in MS-only analyses EDSS [+0.09 points/year, 95% CI: (0.02, 0.015), *P* = 0.012]. Whole group associations of *HDAC9* (+0.05 points/year, 95%CI: 0.01–0.10, *P* = 0.010) and *EVI5* (+0.05 points/year, 95%CI: 0.01–0.10, *P* = 0.03) with annualized change in EDSS were not replicated in MS-only analyses, suggesting over-representation of negative genotypes for both genes within the CIS cohort. In MS-only analysis, *IRX1* was also associated with slower annualized EDSS changes by −0.07 points/year [95% CI: (−0.13, −0.01), *P* = 0.015].

## Discussion

The genetic contribution to MS disability accrual and disease progression remains poorly understood. This 30-year study of a prospectively acquired longitudinal CIS cohort suggests polygenic influences on long-term clinical outcomes in MS, associated with effects on WM (lesions) or GM (cortical lesions or atrophy). HLA-DRB1*1501 was associated with greater WM inflammation (faster accrual and overall lesion volume and number, as well as microstructural changes) and higher relapse rates in MS subjects. *PVRL2* was associated with more cortical lesions, greater 30-year EDSS, greater annualized EDSS changes over time, and increased risk of SPMS. In contrast*, IRX1* was linked with preserved 30-year brain volumes (GMF, MEDW and TVW), reduced risk of cortical lesions, lower 30-year EDSS and in MS patients, lower risk of SPMS and slower EDSS worsening.

Our HLA-DRB1*1501 findings are consistent with cross-sectional work reporting associations with greater WM inflammation,^18–20^ greater changes in normal-appearing WM MTR^18,21^ and longitudinal reports of higher T2 lesion volume in primary progressive MS (PPMS)^21^ and greater T2 lesion volume increase in relapse-onset MS.^9^ No association was seen with cortical lesions, as in a *post mortem* study of younger MS cases^22^ and *in vivo* in a Japanese cohort.^23^ However, this may reflect lower sensitivity detecting cortical lesions compared with WM lesions using MRI,^24^ and genetic differences between ethnicities. In this study, HLA-DRB1*1501 was associated with cross-sectional reduced GMF at 30-years, but this was not significant after adjusting for WM lesion volume, and no association was seen with longitudinal brain atrophy measurements.

Clinically, the HLA-DRB1*1501 allele had a modest positive effect on relapse rates in MS patients, as did *AHI1,* another MS-risk gene associated with greater annualized change in WM lesions. Both have previously been associated with relapse risk in children, AHI1 directly influencing relapse risk^25^ and HLA-DRB1*1501 by modifying the association of vitamin D levels with relapse rate.^26^ While HLA-DRB1*1501 may be associated with earlier onset of cognitive impairment,^17^ we did not find an effect on 30-year cognitive outcomes. HLA-DRB1*1501 has been associated with greater annualized change in EDSS,^9^ but the present results suggest that in the longer term this effect is overtaken by disability linked with other genetic factors. This would be consistent with a functional dichotomy of genetic influences on MS susceptibility, relapses and relapse-associated disability, and in the longer-term a PPMS phenotype and disability accrual independent of relapses.^3, 7^

*PVRL2* encodes a plasma membrane glycoprotein allowing CNS cellular entry and transmission of human herpes viruses.^27,28^ Epstein-Barr virus (HHV-4) and HHV-6, have been implicated as potential viral inflammatory drivers of MS,^29–31^ and latent reactivation may facilitate disease progression.^32^*PVRL2* is also involved in co-inhibitory signalling networks that impair normal regulation of T-cell activity leading to disease progression in experimental autoimmune encephalomyelitis.^33^*PVRL2* has been linked with MSSS in nearly 2000 patients with clinically definite MS and mean disease duration of ∼13 years.^34^ Another study showed no effect of *PVRL2* on long-term EDSS but compared ‘benign’ RRMS (EDSS ≤ 3 and disease duration >20 years) with ‘malignant’ PPMS (EDSS > 6) rather than SPMS.^35^*PVRL2* was also one of several genetic variants selected by machine learning to derive a genetic model of MS severity which predicted disability accrual.^36^

*IRX1* functions in embryonic development of the nervous system,^37,38^ and cell cycle regulation.^39^*IRX1* has not previously been linked with cortical lesions or disability in MS. A previous GWAS linked *IRX1* with brain parenchymal volume in MS but did not reach genome-wide significance and reported no effect on disability.^3^ This may be due to shorter disease duration (range 10.7–12.9 years). Whilst phenotypic separation by EDSS begins between 5 and 10 years following onset (*Figure 1A*), confirming an SPMS diagnosis is challenging and often requires sustained disease progression over several years.^40^ In this cohort, of 15 people with SPMS at 30 years, 10 (67%) transitioned to SPMS between 20 and 30 years and would have been classified with RRMS in shorter-duration studies.

### Limitations

This cohort is uniquely placed to explore potential genetic signals with long-term disease severity, being deeply phenotyped, having a homogenous 30-year disease duration, and longitudinal clinical and MRI measures. While the results build upon previous findings, this is an exploratory study and associations not already established in the literature should be considered again in validation studies.

We selected genes through a literature review and are mindful that this could bias results towards what is already expected. Due to limited sample size, we assumed a dominant genetic model to maximize statistical power^41^ and restricted SNP interrogation significantly compared with exome sequencing or GWAS approaches, which although unbiased would require substantially larger multi-centre cohorts for longitudinal study (e.g. 12 584 cases in Ref. 6 and 1813 cases in Ref. 7) and would therefore be constrained to only widely acquired MRI measures, essentially meaning WM lesion loads. As this was an exploratory study, we have not adjusted for multiple comparisons to maximize sensitivity and minimize type II errors. Bonferroni correction for variant number (0.05/27), as in exome sequencing or GWAS, would change the *P*-value threshold to <1.85 × 10^−3^. Only the association of HLA-DRB1*1501 with 30-year WM lesion volume would remain significant (*P* = 1.27 × 10^−3^), with a borderline association for *PVRL2* (*P* = 2.02 × 10^−3^) with 30-year EDSS in MS participants.

Although we have postulated the mechanisms by which the variants of interest in this study may exert phenotypic influence, their exact functionality and pathogenicity remain to be determined, particularly as they are not located within the exon of their associated genes. Rs3135388 tags HLA-DRB1*1501 through linkage disequilibrium,^42^ whilst rs4866550 (*IRX1*) is intergenic and rs4803766 (*PVRL2*) is intronic. The majority of disease associated variants identified in GWAS are in non-coding regions.^43^ Intergenic variants such as rs4866550 (*IRX1*) can influence gene expression by altering activity at promoter or enhancer sites through effects on chromatin states.^44,45^ Intronic SNPs such as rs4803766 (*PVRL2*) can alter mRNA splicing, augment gene expression and influence mRNA transport and chromatin assembly.^46^

Indeed, individual SNPs may have minimal functional impact but be in linkage disequilibrium with other variants to form a haplotype that exerts a functional genetic influence.^47^

Since completing genotyping for this study, two large-scale GWAS have implicated previously unnoticed SNPs with age-related MS severity (rs10191329)^6^ and in sex-stratified analyses of MS severity (rs10967273 and rs698805),^7^ and it is likely that they will not be the last to be found. It would be of great interest to further explore the pathological mechanisms underpinning these associations using a similar framework to this study. For example, rs10191329 was associated with cortical lesions, mirroring associations of *PVRL2* (rs4803766) and *IRX1* (rs4866550) observed in this study, and further supporting cortical pathology as a significant correlate of progressive disease. We would therefore hypothesize that all three SNPs would also be associated with brain atrophy. Unfortunately, post-hoc analysis of these novel SNPs was not possible here, as the genotyping array used (Illumina Infinium GSA) did not include these variants within its fixed markers.

Variability and underestimation of outcome measures may have limited ability to detect genetic associations with smaller effects. As genetic testing was only undertaken in participants attending at 30-year follow-up, this study did not include the most disabled SPMS participants, who were unable to attend, or participants who died from MS; in whom, disability accrual and atrophy were greatest at earlier study timepoints.^11^ Compared to the original cohort, participants in this study were less likely to have developed SPMS (25% versus 41%) and took longer to do so (mean 19.6 years versus 15.3 years).^10,11^ Mild relapses may not have been recalled due to lengthy follow-up intervals and many cortical lesions remain undetected on 3T MRI, which may explain clearer associations with binary presence (‘Yes/No’) of cortical lesions. Changes in MRI scanner system and field strength over 30 years will have increased variability in WM lesion and linear brain atrophy measures (see Ref. 11). Measurement noise, particularly in early scans, and differing longitudinal MEDW and TVW trajectories^11^ may explain why *IRX1* was associated with all 30-year cross-sectional brain volume measures but only TVW longitudinally.

Despite these limitations, we have replicated associations of HLA-DRB1*1501 with WM lesion measures,^9,18–21^ providing reassurance that this cohort is sufficiently large and clinically diverse to detect substantial genetic effects.

## Conclusion

The results of this exploratory study are consistent with polygenic influences on long-term MS outcomes mediated by different pathological mechanisms and suggest that genetic influences on GM pathology have the most clinical impact in the long term. These findings, and potential genetic associations with long-term disability and SPMS, may be useful in developing holistic models of disease progression but need to be tested in larger cohorts with similar disease durations.

## Supplementary material

Supplementary material is available at *Brain Communications* online.

## Supplementary Material

fcad255_Supplementary_Data

## Data Availability

Anonymized data, not published in the article, can be shared on reasonable request from a qualified investigator.
