# Supplementary material for: Genetic influences on disease course and severity, 30 years after a clinically isolated syndrome
Source: Brain Commun. 2023 Oct 4;5(5):fcad255. doi: 10.1093/braincomms/fcad255 (PMC10576246; doi:10.1093/braincomms/fcad255)
Supplement: fcad255_Supplementary_Data [file fcad255_supplementary_data.docx]

**Supplementary Table 1 - SNP selection by known associations with inflammation, neurodegeneration and clinical outcomes in multiple sclerosis**

| **SNP (alleles)** | **Minor Allele** | **CHR** | **Position** | **Variant****Function** | **Call-rate** | **MAF** | **HWE p-value** | **Gene** | **Associations** | | | | |
| --- | --- | --- | --- | --- | --- | --- | --- | --- | --- | --- | --- | --- | --- |
|  |  |  |  |  |  |  |  |  | **Inflammation** | **Neurodegeneration** | | **Clinical** | |
| rs3135388(T/C) | T | 6 | 32413051 | Intergenic | 1.000 | 0.24 | 0.75 | *HLA-DRB1*1501* | Increased WM lesion measures.^1–3^ Inflammation within both cortical NAGM and lesional GM, and more extensive cortical lesions in younger multiple sclerosis patients.^4^  Greater frequency and number of cortical lesions.^5^ | Whole brain atrophy and GM atrophy.^1,2^ | | Lower age of onset^1,6^ and worse cognitive impairment.^1^ | |
| rs2743951(A/G) | A | 6 | 29709234 | Intron | 1.000 | 0.39 | 0.29 | *HLA B*4402* | Reduced T2 WM lesion burden.^7^ | | Preserved brain parenchymal fraction^7^ and protective on subcortical GM atrophy.^8^ | | Protective effect on cognition assessed by SDMT.^9^ Protective for both conversion to multiple sclerosis and relapses.^10^ |
| rs4713274(G/C) | G | 6 | 29937493 | Intergenic | 0.951 | 0.16 | 0.69 | *HLA A*02:01* | Weak protective effect on new WM lesions.^11^ Weak protective effect on T2 WM lesion volume in DRB1*1501-ve patients.^9^ | |  | | Associated with weak protective effects on disability (EDSS and MSSS).^11^Decreased hazard ratio for SPMS.^12^ Weak protective effect on SDMT in DRB1*1501 +ve patients.^9^ |
| rs4315313(T/C) | T | 16 | 83086717 | Intron | 0.967 | 0.41 | 0.9 | *CDH13* | WM T2 lesion load.^13^ | |  | |  |
| rs6917747(A/G) | A | 6 | 160402705 | Intron | 0.951 | 0.17 | 0.51 | *IGF2R* | WM T2 Lesion Load.^13^ | |  | |  |
| rs2039485*(A/G) | G | 14 | 32353250 | Intergenic | 0.918 | 0.24 | 3E-11 | *NUBPL* | WM T2 Lesion Load.^13^ | |  | |  |
| rs17208888*(A/G) | A | 6 | 32379506 | Intergenic | 0.984 | 0.13 | 0.015 | *HLA-DRB1 *11:04* | Thoracic cord WM lesions.^14^ | |  | |  |
| rs59655222 (T/C) | C | 1 | 200875897 | Intron | 0.951 | 0.34 | 0.52 | *C1orf106, KIF21B* | *KIF21B* associated with more extensive GM demyelination.^15^ | |  | | Multiple sclerosis risk variant in IMSGC 2019.^16^ Accelerated development of EDSS 6 in multiple sclerosis patients.^15^ |
| rs2389963(A/G) | G | 7 | 18588445 | Intron | 0.967 | 0.37 | 0.5 | *HDAC* |  | | Histone deacetylase gene variants predict brain volume changes in multiple sclerosis.^17^ | |  |
| rs2074633(T/C) | C | 7 | 19035920 | 3 prime untranslated region | 0.951 | 0.28 | 0.7 | *HDAC9* |  | | Histone deacetylase gene variants predict brain volume changes in multiple sclerosis.^17^ | |  |
| rs1927457*(T/C) | C | 10 | 30008663 | Intron | 0.934 | 0.33 | 0.43 | *SVIL* |  | | Brain parenchymal volume atrophy.^13^ | |  |
| rs11957313(A/G) | A | 5 | 169950394 | Intron | 0.967 | 0.24 | 0.81 | *KCNIP1* |  | | Brain parenchymal volume atrophy.^13^ | |  |
| rs4866550(T/C) | T | 5 | 33083129.0807 | Intergenic | 0.984 | 0.31 | 0.43 | *IRX1* |  | | Brain parenchymal volume atrophy.^13^ | |  |
| rs4473631(T/G) | G | 4 | 174501769 | Intron | 0.951 | 0.29 | 0.52 | *MORF4* |  | | Brain parenchymal volume atrophy.^13^ | |  |
| rs1869410(T/C) | C | 2 | 52573511.8860 | Intergenic | 0.967 | 0.3 | 0.26 | *SOX11* |  | | Brain parenchymal volume atrophy.^13^ | |  |
| rs9486902(T/C) | T | 6 | 108878052 | Intergenic | 0.984 | 0.14 | 0.83 | *FOXO3A* |  | | Brain parenchymal volume atrophy.^13^ | |  |
| rs7104613*(T/C) | T | 11 | 14079931 | Intron | 1.000 | 0.13 | 0.028 | *SPON1* |  | | Reduced deep grey matter volume.^18^ | |  |
| rs77970177*(T/C) | T | 1 | 72962190 | Intergenic | 1.000 | 0.05 | <0.0001 | *GRIN2B* |  | | Reduced deep grey matter volume.^18^ | |  |
| rs17157903*(A/G) | A | 7 | 103628036 | Intron | 0.967 | 0.14 | 0.0033 | *RELN* |  | |  | | Age of multiple sclerosis onset.^13^ |
| rs1386330*(A/G) | G | 11 | 87819427 | Intron | 0.918 | 0.19 | 0.4 | *RAB38* |  | |  | | Age of multiple sclerosis onset.^13^ |
| rs1557351(A/G) | G | 18 | 54752314 | TF binding site | 0.951 | 0.15 | 0.79 | *WDR7* |  | |  | | Age of multiple sclerosis onset.^13^ |
| rs694739(T/C) | C | 11 | 64097233 | Intergenic | 0.967 | 0.35 | 0.94 | *PRDX5, CCDC88B* |  | |  | | Increased risk of conversion to multiple sclerosis.^10^ |
| rs802734*(A/G) | A | 6 | 128278798 | Intergenic | 0.934 | 0.23 | 0.44 | *PTPRK, THEMIS* |  | |  | | GG phenotype associated with increased risk of conversion to multiple sclerosis.^10^ |
| rs1323292(A/G) | A | 1 | 192541021 | Intergenic | 0.984 | 0.23 | 0.44 | *RGS1, RGS21* |  | |  | | AG+GG phenotype associated with increased risk of conversion to multiple sclerosis.^10^ |
| rs12988804*(T/C) | T | 2 | 170117811 | Intron | 0.934 | 0.27 | 0.42 | *LRP2* |  | |  | | First variant validated at genome wide significance as marker of multiple sclerosis severity (relapse rate) with hazard ratio (HR) 2.18.^19,20^ |
| rs9277561(A/G) | G | 6 | 33056749 | Intergenic | 0.967 | 0.25 | 0.9 | *HLA-DPB1* |  | |  | | Susceptibility variant,^16^ also linked to increased relapse risk.^10^ |
| rs11154801(T/G) | T | 6 | 135739355 | Intron | 0.951 | 0.40 | 0.3 | *AHI1* |  | |  | | Known multiple sclerosis risk allele.^16^Also associated with increased relapse rate.^21^ |
| rs11810217(A/G) | A | 1 | 93148377 | Intron | 0.984 | 0.33 | 0.7 | *EVI5* |  | |  | | Multiple sclerosis risk variant in IMSGC 2019.^16^Associated with increased relapse risk.^22^ |
| rs2300603(T/C) | C | 14 | 76005557 | Intron | 0.951 | 0.22 | 0.41 | *BATF* |  | |  | | Multiple sclerosis risk variant in IMSGC 2019.^16^Associated with increased relapse risk.^22^ |
| rs7775055*(A/G) | G | 6 | 32657916 | TF binding site | 0.980 | 0.02 | 0.9 | *HLA DRB1*08* |  | |  | | More prevalent among RRMS patients and was associated with the lower rate of relapse, degree of disability and IgG index.^23^75% lower odds that RRMS will change to a progressive course multiple sclerosis.^24^ |
| rs2283792*(T/G) | G | 22 | 22131125 | Intron | 0.934 | 0.46 | 0.54 | *MAPK1* |  | |  | | One of seven non-HLA SNPs predicting annualised ΔEDSS with dose-response when incorporated into a cumulative genetic risk score.^10^ |
| rs2546890(T/C) | C | 5 | 158759900 | Non coding transcript exon | 1.000 | 0.43 | 0.25 | *LOC285626* |  | |  | | One of seven non-HLA SNPs predicting annualised ΔEDSS with dose-response when incorporated into a cumulative genetic risk score.^10^ |
| rs10516537(T/G) | T | 4 | 107628071 | Regulatory region | 0.967 | 0.17 | 0.78 | *DKK2* |  | |  | | Disability (MSSS).^13^ |
| rs4803766(A/G) | A | 19 | 45371168 | Intron | 0.967 | 0.44 | 0.81 | *PVRL2* |  | |  | | Disability (MSSS).^25^ |
| rs752092(A/G) | G | 15 | 101781934 | Intron | 0.951 | 0.37 | 0.59 | *CHSY1* |  | |  | | Disability (MSSS).^13^ |
| rs12638253(A/G) | A | 3 | 156626091 | Intron | 1.000 | 0.47 | 0.87 | *FLJ16641* |  | |  | | Disability (MSSS).^13^ |
| rs10518025(A/G) | G | 4 | 68064440 | Regulatory region | 0.967 | 0.15 | 0.17 | *CENPC1* |  | |  | | Disability (MSSS).^13^ |
| rs12142240(A/G) | G | 1 | 46747301 | Intron | 0.951 | 0.26 | 0.26 | *LRRC41* |  | |  | | Disability (MSSS).^13^ |

SNP – single nucleotide polymorphism, CHR – Chromosome, MAF – minor allele frequency, HWE – Hardy-Weinberg equilibrium, WM – white matter, GM – grey matter, NAGM – normal appearing grey matter, MS – multiple sclerosis, SDMT – Symbol digit Modalities Test, EDSS – expanded disability status scale, MSSS – multiple sclerosis severity score, NF – neurofibrillary, RRMS – relapsing-remitting MS. TF – Transcription Factor. Variant function of SNPs as provided in ENSEMBL using Genome Reference Consortium Human Build 37 patch release 13 (GRCh37.p13).

**SNP Selection and genotyping**

Potential variants were identified from GWAS catalog (<https://www.ebi.ac.uk/gwas>), and dbSNP (<https://www.ncbi.nlm.nih.gov/snp>) through searches for “multiple sclerosis” with either “lesion”, “inflammation”, “neurodegeneration”, volume”, “atrophy”, “relapse”, “onset”, “severity”, “phenotype”, or “disability” using an “AND” Boolean operator. A Pubmed search was undertaken with the same search targets: ("multiple sclerosis"[Title]) AND ("gene"[Title] OR "SNP"[Title] OR "genetic"[Title] OR "genome"[Title] OR "HLA"[Title]) AND ("lesion"[Title] OR "inflammation"[Title] OR "neurodegeneration"[Title] OR "volume"[Title] OR "atrophy"[Title] OR "severity"[Title] OR "phenotype"[Title] OR "disability" [Title] OR "onset"[Title])).

Where tagging SNPs for potential genetic variants were not provided within the study article or supplementary material, these were identified, if available, through cross-referencing with GWAS catalog and dbSNP databases. Thirty-eight genetic variants, including *HLA-DRB1*1501*, were identified with tagging SNPs identifiable using the Infinium Global Screening Array-24 v3.0 Beadchip from Illumina (San Diego, CA, USA). This included variants from several GWAS studies, with suggestive genetic associations that did not reach genome wide significance, and longitudinal cohort studies, of shorter follow-up than our cohort, where phenotypic associations were identified either with SNPs individually or within cumulative genetic risk scores. Eleven SNPs were excluded (*) from analysis if minor allele frequency (MAF) was <0.05, call rate <95%, if there was significant deviation from Hardy-Weinberg equilibrium (p<0.05) or individual heterozygosity rate >3 standard deviations from mean.^28^ MAF threshold was set at <0.05 to cautiously limit rare genetic variants, given the relatively small cohort size.

**Supplementary Table 2 – All significant (p<0.05) SNP associations at 30-year follow-up in all participants (MS and CIS)**

|  | | **Gene, *SNP (allele)*** | | | | | | | | | | | | | | | |
| --- | --- | --- | --- | --- | --- | --- | --- | --- | --- | --- | --- | --- | --- | --- | --- | --- | --- |
| **Outcome at 30-year**  **Follow Up** | | ***HLA-DRB1*1501* *rs3135388 (T)*** | ***HDAC***  ***rs2389963 (G)*** | ***LOC285626***  ***rs2546890 (C)*** | ***BATF***  ***rs2300603 (C)*** | ***PVRL2***  ***rs4803766 (A)*** | ***IRX1***  ***rs4866550 (T)*** | ***KCNIP1***  ***rs11957313 (A)*** | ***HDAC9***  ***rs2074633 (C)*** | ***CHSY1***  ***rs752092 (G)*** | ***WDR7***  ***rs1557351 (G)*** | ***FLJ16641***  ***rs12638253 (A)*** | ***EVI5***  ***rs11810217 (A)*** | ***CDH13***  ***rs4315313 (T)*** | ***RGS1, RGS21***  ***rs1323292 (G)*** | **DKK2**  **rs10516537 *(T)*** | ***IGF2R***  ***rs6917747 (A)*** |
| **Inflammation** |  |  |  |  |  |  |  |  |  |  |  |  |  |  |  |  |  |
| **White Matter** |  |  |  |  |  |  |  |  |  |  |  |  |  |  |  |  |  |
| WMLs *(n)* | Estimate  95% CI | 54.99  3.60 to 104.79^b^ |  |  |  |  |  |  |  |  |  |  |  |  |  |  |  |
|  | R^2^ | 0.15 |  |  |  |  |  |  |  |  |  |  |  |  |  |  |  |
|  | p-value | 0.021 |  |  |  |  |  |  |  |  |  |  |  |  |  |  |  |
| WMLV *(ml)* | Estimate  95% CI | 11.60 5.49 to 18.29^b^ |  |  |  |  |  |  |  |  |  |  |  |  |  |  |  |
|  | R^2^ | 0.28 |  |  |  |  |  |  |  |  |  |  |  |  |  |  |  |
|  | p-value | **0.001**** |  |  |  |  |  |  |  |  |  |  |  |  |  |  |  |
| WML MTR *(pu)* | Estimate  95% CI | -1.17  -0.24 to -2.26^b^ |  |  |  |  |  |  |  |  |  |  |  |  |  |  |  |
|  | R^2^ | 0.18 |  |  |  |  |  |  |  |  |  |  |  |  |  |  |  |
|  | p-value | 0.013 |  |  |  |  |  |  |  |  |  |  |  |  |  |  |  |
| NAWM MTR *(pu)* | Estimate  95% CI | -0.85  -0.14 to -1.66^b^ |  |  |  |  |  |  |  |  |  |  |  |  |  |  |  |
|  | R^2^ | 0.20 |  |  |  |  |  |  |  |  |  |  |  |  |  |  |  |
|  | p-value | 0.021 |  |  |  |  |  |  |  |  |  |  |  |  |  |  |  |
| **Grey Matter** |  |  |  |  |  |  |  |  |  |  |  |  |  |  |  |  |  |
| GM lesions (*n)* | Estimate  95% CI |  |  |  | -0.81  -0.20 to -1.41^b^ | 0.83  0.08 to 1.66^b^ |  |  |  |  |  |  |  |  |  |  |  |
|  | R^2^ |  |  |  | 0.14 | 0.16 |  |  |  |  |  |  |  |  |  |  |  |
|  | p-value |  |  |  | 0.029 | 0.042 |  |  |  |  |  |  |  |  |  |  |  |
| GM lesions *(Y/N?)* | OR  95% CI |  |  |  | 0.09 0.01 to 0.81 | 7.23  1.13 to 46.21 | 0.22  0.05 to 0.99 |  |  |  |  |  |  |  |  |  |  |
|  | R^2^ |  |  |  | 0.37 | 0.38 | 0.34 |  |  |  |  |  |  |  |  |  |  |
|  | p-value |  |  |  | 0.032 | 0.037 | 0.049 |  |  |  |  |  |  |  |  |  |  |
| cGM MTR gradient  (pu/band) | Estimate  95% CI |  |  |  |  |  | 0.15  0.02 to 0.29^b^ |  |  |  |  |  |  |  |  |  |  |
|  | R^2^  p-value |  |  |  |  |  | 0.23  0.019 |  |  |  |  |  |  |  |  |  |  |
| **Brain Volume** |  |  |  |  |  |  |  |  |  |  |  |  |  |  |  |  |  |
| GMF (%) | Estimate  95% CI | -0.62  0.00 to -1.36^b^ |  |  |  |  | 0.76  0.28 to 1.29^b^ | -0.72  -0.12 to -1.55^b^ | -0.72  -0.10 to  -1.37^b^ |  |  |  |  |  |  |  |  |
|  | R^2^ | 0.41 |  |  |  |  | 0.42 | 0.44 | 0.41 |  |  |  |  |  |  |  |  |
|  | p-value | 0.03 |  |  |  |  | **0.008*** | 0.016 | 0.014 |  |  |  |  |  |  |  |  |
| MEDW *(mm)* | Estimate |  |  |  |  |  | 0.28 | -0.25 |  |  |  |  |  |  |  |  |  |
|  | 95% CI  R^2^ |  |  |  |  |  | 0.03 to 0.46^b^  0.14 | -0.02 to -0.49  0.14 |  |  |  |  |  |  |  |  |  |
|  | p-value |  |  |  |  |  | 0.042 | 0.035 |  |  |  |  |  |  |  |  |  |
| TVW *(mm)* | Estimate |  |  |  |  |  | -1.82 |  |  | -1.71 |  |  |  |  |  |  |  |
|  | 95% CI  R^2^ |  |  |  |  |  | -0.52 to -3.22^b^  0.22 |  |  | -0.18 to -3.06^b^  0.23 |  |  |  |  |  |  |  |
|  | p-value |  |  |  |  |  | 0.014 |  |  | 0.027 |  |  |  |  |  |  |  |
| **Clinical** |  |  |  |  |  |  |  |  |  |  |  |  |  |  |  |  |  |
| **Neuropsychology** |  |  |  |  |  |  |  |  |  |  |  |  |  |  |  |  |  |
| BVMTR-z | Estimate  95% CI |  |  |  |  |  |  |  |  |  | 1.10  0.35 to 1.85 | -0.81  -0.00 to -1.63 | -0.85  -0.16 to -1.54^b^ | -0.83  -0.50 to -1.60^b^ | -0.79  -0.12 to -1.45 |  |  |
|  | R^2^  p-value |  |  |  |  |  |  |  |  |  | 0.27  **0.005*** | 0.18  0.050 | 0.23  0.017 | 0.19  0.032 | 0.21  0.021 |  |  |
| CVLT-z | Estimate  95% CI |  | 0.634  0.03 to 1.24 |  |  |  |  |  |  |  |  |  |  |  |  |  |  |
|  | R^2^  p-value |  | 0.43  0.041 |  |  |  |  |  |  |  |  |  |  |  |  |  |  |
| SDMT-z | Estimate  95% CI |  |  |  | 0.715  0.02 to 1.42 |  |  |  | 0.736  0.05 to 1.42 |  |  |  |  | -0.70  -0.04 to -1.37 |  |  |  |
|  | R^2^  p-value |  |  |  | 0.23  0.046 |  |  |  | 0.24  0.035 |  |  |  |  | 0.24  0.038 |  |  |  |
| BICAMS Total | Estimate  95% CI |  |  |  | 15.23  4.94 to 26.31^b^ |  |  |  |  |  |  |  |  |  |  |  |  |
|  | R^2^  p-value |  |  |  | 0.23  0.016 |  |  |  |  |  |  |  |  |  |  |  |  |
| FSS | Estimate  95% CI |  |  |  |  |  | -7.98  -0.10 to -15.87 ^b^ |  | 12.92 3.05 to 18.81 |  |  |  |  |  |  | -9.62  -0.37 to -18.86 |  |
|  | R^2^  p-value |  |  |  |  |  | 0.26  0.047 |  | 0.23  **0.008*** |  |  |  |  |  |  | 0.18  0.042 |  |
| PASAT-3 | Estimate  95% CI |  |  |  |  |  |  |  |  |  |  |  |  |  |  |  | 7.41  0.20 to 14.62 |
|  | R^2^  p-value |  |  |  |  |  |  |  |  |  |  |  |  |  |  |  | 0.20  0.044 |
| **Disability** |  |  |  |  |  |  |  |  |  |  |  |  |  |  |  |  |  |
| 9HPT-ND *(s)* | Estimate  95% CI |  |  | 6.17  2.17 to 10.39^b^ |  |  | -6.38  -2.32 to -11.14 |  |  |  |  |  |  |  |  |  |  |
|  | R^2^  p-value |  |  | 0.32  0.013 |  |  | 0.32  **0.009*** |  |  |  |  |  |  |  |  |  |  |
| EDSS | Estimate  95% CI |  |  |  |  | 1.72  0.49 to 2.93^b^ | -1.35  -0.29 to -2.48^b^ |  |  |  |  |  |  |  |  |  |  |
|  | R^2^  p-value |  |  |  |  | 0.25  0.013 | 0.22  0.026 |  |  |  |  |  |  |  |  |  |  |
| **Phenotype** |  |  |  |  |  |  |  |  |  |  |  |  |  |  |  |  |  |
| Progression to SPMS *(Y/N?)* | OR  95% CI |  |  |  |  | 12.25 1.15 to 23.10^b^ |  |  |  |  |  |  |  |  |  |  |  |
|  | R^2^  p-value |  |  |  |  | 0.31  0.031 |  |  |  |  |  |  |  |  |  |  |  |

OR – Odds Ratio, CI – confidence interval, SNP – single nucleotide polymorphism, WML – white matter lesion, WMLV – white matter lesion volume, NAWM – normal appearing white matter, MTR – magnetisation transfer ratio, pu – percentage units, GM – grey matter, cGM – cortical grey matter, GMF – grey matter fraction, BVMTR-z - Brief Visuospatial Memory Test–Revised z-score adjusted for age, sex and education, BICAMS – brief international cognitive assessment for MS, CVLT-z – California verbal learning test z-score, FSS – fatigue severity scale, PASAT-3 – paced serial auditory test-3, SDMT-z – Symbol digit Modalities Test z-score, 9HPT-D – 9 hole peg test dominant hand, 9HPT-ND – 9 hole peg test non-dominant hand, EDSS -expanded disability status scale, MS – multiple sclerosis, SPMS – Secondary progressive MS,

^b^ – Bootstrapped confidence intervals using 1000 replicates

*** p-value < 0.01**

**** p < 1.85 x 10-3** *(*Bonferroni correction for number of variants = 0.05/27 variants)

**Supplementary Table 3 – All significant (p<0.05) SNP associations at 30-year follow-up in participants with MS only (excluding CIS)**

| **Outcome at 30-year**  **Follow Up** | |  | **Gene, *SNP (allele)*** | | | | | | | | | | | | |
| --- | --- | --- | --- | --- | --- | --- | --- | --- | --- | --- | --- | --- | --- | --- | --- |
|  |  | ***HLA-DRB1*1501* *rs3135388 (T)*** | ***HLA B*4402***  ***rs2743951 (A)*** | ***BATF***  ***rs2300603 (C)*** | ***PVRL2***  ***rs4803766 (A)*** | ***IRX1***  ***rs4866550 (T)*** | ***HDAC9***  ***rs2074633 (C)*** | ***WDR7***  ***rs1557351 (G)*** | ***CHSY1***  **rs752092 (G)** | ***EVI5***  ***rs11810217 (A)*** | ***LRRC41***  ***rs12142240 (G)*** | ***CENPC1***  ***rs10518025 (G)*** | ***FLJ16641***  ***rs12638253 (A)*** | **DKK2**  **rs10516537 *(T)*** | ***IGF2R***  ***rs6917747 (A)*** |
| **Inflammation** |  |  |  |  |  |  |  |  |  |  |  |  |  |  |  |
| **White Matter** |  |  |  |  |  |  |  |  |  |  |  |  |  |  |  |
| WMLs *(n)* | Estimate  95% CI | 57.94  1.03 to 114.84 |  |  |  |  |  |  |  |  |  |  |  |  |  |
|  | R^2^ | 0.13 |  |  |  |  |  |  |  |  |  |  |  |  |  |
|  | p-value | 0.046 |  |  |  |  |  |  |  |  |  |  |  |  |  |
| WMLV *(ml)* | Estimate  95% CI | 11.05  3.18 to 18.87^b^ |  |  |  |  |  |  |  |  |  |  |  |  |  |
|  | R^2^ | 0.22 |  |  |  |  |  |  |  |  |  |  |  |  |  |
|  | p-value | 0.011 |  |  |  |  |  |  |  |  |  |  |  |  |  |
| WML MTR *(pu)* | Estimate  95% CI | -1.37  -0.41 to  -2.46^b^ |  |  |  |  |  |  |  |  |  |  |  |  |  |
|  | R^2^ | 0.26 |  |  |  |  |  |  |  |  |  |  |  |  |  |
|  | p-value | 0.014 |  |  |  |  |  |  |  |  |  |  |  |  |  |
| NAWM MTR *(pu)* | Estimate  95% CI | -0.816  -0.03 to -1.63^b^ |  |  |  |  |  |  |  |  |  |  | -1.087  -0.08 to -2.07^b^ |  |  |
|  | R^2^ | 0.22 |  |  |  |  |  |  |  |  |  |  | 0.25 |  |  |
|  | p-value | 0.045 |  |  |  |  |  |  |  |  |  |  | 0.022 |  |  |
| **Grey Matter** |  |  |  |  |  |  |  |  |  |  |  |  |  |  |  |
| cGM MTR *(pu)* | Estimate  95% CI |  | 0.584  0.06 to 1.17^b^ |  |  |  |  |  |  |  |  |  | -0.712  -0.01 to  -1.69^b^ |  |  |
|  | R^2^ |  | 0.29 |  |  |  |  |  |  |  |  |  | 0.29 |  |  |
|  | p-value |  | 0.049 |  |  |  |  |  |  |  |  |  | 0.034 |  |  |
| Cortical lesions (*n)* | Estimate  95% CI |  |  | -1.11  -0.18 to  -2.05^b^ | 1.13  0.15 to 2.12^b^ |  |  |  |  |  |  |  |  |  |  |
|  | R^2^ |  |  | 0.19 | 0.16 |  |  |  |  |  |  |  |  |  |  |
|  | p-value |  |  | 0.021 | 0.047 |  |  |  |  |  |  |  |  |  |  |
| Cortical lesions *(Y/N?)* | OR  95% CI |  |  | 0.07  0.01 to 0.65 | 9.85  1.20 to 81.04 | 0.14  0.03 to 0.73 |  |  |  | 5.70  1.07 to 30.30 |  |  |  |  |  |
|  | R^2^ |  |  | 0.43 | 0.38 | 0.39 |  |  |  | 0.34 |  |  |  |  |  |
|  | p-value |  |  | 0.020 | 0.033 | 0.020 |  |  |  | 0.041 |  |  |  |  |  |
| cGM MTR gradient  (pu/band) | Estimate  95% CI |  |  |  |  |  | -0.19  -0.03 to  -0.37^b^ |  |  |  |  |  |  |  |  |
|  | R^2^  p-value |  |  |  |  |  | 0.26  0.031 |  |  |  |  |  |  |  |  |
| **Brain Volume** |  |  |  |  |  |  |  |  |  |  |  |  |  |  |  |
| GMF (%) | Estimate  95% CI |  |  |  |  | 0.88  0.28 to 1.64^b^ | -1.18  -0.48 to  -2.08^b^ | -1.03  -0.18 to  -2.20^b^ |  |  |  |  |  |  |  |
|  | R^2^ |  |  |  |  | 0.36 | 0.45 | 0.38 |  |  |  |  |  |  |  |
|  | p-value |  |  |  |  | 0.024 | **0.002*** | 0.026 |  |  |  |  |  |  |  |
| MEDW *(mm)* | Estimate |  |  |  |  | 0.31 |  |  |  |  |  |  |  |  |  |
|  | 95% CI  R^2^ |  |  |  |  | 0.06 to 0.57  0.19 |  |  |  |  |  |  |  |  |  |
|  | p-value |  |  |  |  | 0.019 |  |  |  |  |  |  |  |  |  |
| TVW *(mm)* | Estimate |  |  |  |  | -2.03 |  |  | -2.42 |  |  |  |  |  |  |
|  | 95% CI  R^2^ |  |  |  |  | -0.32 to  -3.87^b^  0.19 |  |  | -0.50 to  -4.22^b^  0.23 |  |  |  |  |  |  |
|  | p-value |  |  |  |  | 0.035 |  |  | 0.023 |  |  |  |  |  |  |
| **Clinical** |  |  |  |  |  |  |  |  |  |  |  |  |  |  |  |
| **Neuropsychology** |  |  |  |  |  |  |  |  |  |  |  |  |  |  |  |
| BVMTR-z | Estimate  95% CI |  |  |  | -1.09  -0.10 to  -2.08 |  |  | 1.21  0.37 to 2.06^b^ |  | -1.08  -0.21 to  -2.06^b^ | 0.97  0.06 to 1.93^b^ |  |  |  |  |
|  | R^2^  p-value |  |  |  | 0.15  0.033 |  |  | 0.28  0.023 |  | 0.30  0.021 | 0.26  0.031 |  |  |  |  |
| CVLT-z | Estimate  95% CI |  |  |  |  |  |  |  |  |  |  | -0.94  -0.20 to  -1.68 |  |  |  |
|  | R^2^  p-value |  |  |  |  |  |  |  |  |  |  | 0.59  0.015 |  |  |  |
| SDMT-z | Estimate  95% CI |  |  |  |  |  |  |  |  |  |  | -1.23  -0.12 to  -2.00^b^ | -1.12  -0.26 to  -1.98^b^ |  |  |
|  | R^2^  p-value |  |  |  |  |  |  |  |  |  |  | 0.38  **0.006*** | 0.33  0.013 |  |  |
| BICAMS Total | Estimate  95% CI |  |  |  |  |  |  |  |  | -15.63  -1.23 to  -32.13 ^b^ |  | -24.97  -7.70 to  -42.24 |  |  |  |
|  | R^2^  p-value |  |  |  |  |  |  |  |  | 0.33  0.046 |  | 0.39  **0.006*** |  |  |  |
| FSS | Estimate  95% CI |  |  |  | 13.77  2.52 to 25.03 | -14.12  -4.62 to  -23.61 | 13.69  3.91 to 23.48 |  |  |  |  |  | 12.52  0.90 to 24.15 | -11.49  -1.05 to  -22.61^b^ |  |
|  | R^2^  p-value |  |  |  | 0.24  0.018 | 0.28  **0.008*** | 0.26  **0.008*** |  |  |  |  |  | 0.20  0.036 | 0.19  0.047 |  |
| PASAT-3 | Estimate  95% CI |  |  |  |  |  |  |  |  |  |  |  |  |  | 11.84  3.67 to 18.81^b^ |
|  | R^2^  p-value |  |  |  |  |  |  |  |  |  |  |  |  |  | 0.25  0.013 |
| **Disability** |  |  |  |  |  |  |  |  |  |  |  |  |  |  |  |
| 9HPT-ND *(s)* | Estimate  95% CI |  |  | -7.10  -0.70 to  -13.97^b^ |  | -8.78  -3.70 to  -14.16 |  |  |  |  |  |  |  |  | 7.45  -0.15 to 16.05^b^ |
|  | R^2^  p-value |  |  | 0.36  0.031 |  | 0.40  **0.006*** |  |  |  |  |  |  |  |  | 0.36  0.044 |
| 9HPT-D *(s)* | Estimate  95% CI |  |  |  |  |  |  |  |  |  |  |  | 11.93  3.07 to 24.18^b^ |  |  |
|  | R^2^  p-value |  |  |  |  |  |  |  |  |  |  |  | 0.32  0.036 |  |  |
| EDSS | Estimate  95% CI |  |  |  | 2.60  1.30 to 3.87^b^ | -2.12  -0.87 to  -3.44^b^ |  |  |  |  |  |  |  |  |  |
|  | R^2^  p-value |  |  |  | 0.33  **0.002*** | 0.29  **0.005*** |  |  |  |  |  |  |  |  |  |
| **Phenotype** |  |  |  |  |  |  |  |  |  |  |  |  |  |  |  |
| Progression to SPMS *(Y/N?)* | OR  95% CI |  |  |  | 15.16  1.16 to 26.67^b^ | 0.19  0.04 to 0.92 |  |  |  |  |  |  |  |  |  |
|  | R^2^  p-value |  |  |  | 0.31  0.029 | 0.24  0.042 |  |  |  |  |  |  |  |  |  |

OR – Odds Ratio, CI – confidence interval, SNP – single nucleotide polymorphism, WML – white matter lesion, WMLV – white matter lesion volume, NAWM – normal appearing white matter, MTR – magnetisation transfer ratio, pu – percentage units, GM – grey matter, cGM – cortical grey matter, GMF – grey matter fraction, BVMTR-z - Brief Visuospatial Memory Test–Revised z-score adjusted for age, sex and education, BICAMS – brief international cognitive assessment for MS, CVLT-z – California verbal learning test z-score, FSS – fatigue severity scale, PASAT-3 – paced serial auditory test-3, SDMT-z – Symbol digit Modalities Test z-score, 9HPT-D – 9 hole peg test dominant hand, 9HPT-ND – 9 hole peg test non-dominant hand, EDSS -expanded disability status scale, MS – multiple sclerosis, SPMS – Secondary progressive MS,

^b^ – Bootstrapped confidence intervals using 1000 replicates

*** p-value < 0.01**

NB: Bonferroni correction for number of variants = 0.05/27 variants = p < 1.85 x 10^-3^

**Reference List**

1. Okuda DT, Srinivasan R, Oksenberg JR, et al. Genotype-phenotype correlations in multiple sclerosis: HLA genes influence disease severity inferred by 1HMR spectroscopy and MRI measures. *Brain*. 2009;132(1):250-259. doi:10.1093/brain/awn301

2. Zivadinov R, Uxa L, Bratina A, et al. HLA-DRB1*1501, -DQB1*0301, -DQB1*0302, -DQB1*0602, and -DQB1*0603 Alleles are Associated With More Severe Disease Outcome on Mri in Patients With Multiple Sclerosis. *Int Rev Neurobiol*. 2007;79(07):521-535. doi:10.1016/S0074-7742(07)79023-2

3. Horakova D, Zivadinov R, Weinstock-Guttman B, et al. HLA DRB1*1501 is only modestly associated with lesion burden at the first demyelinating event. *J Neuroimmunol*. 2011;236(1-2):76-80. doi:10.1016/j.jneuroim.2011.04.011

4. Yates RL, Esiri MM, Palace J, Mittal A, Deluca GC. The influence of HLA-DRB1*15 on motor cortical pathology in multiple sclerosis. *Neuropathol Appl Neurobiol*. 2015;41(3):371-384. doi:10.1111/nan.12165

5. Shinoda K, Matsushita T, Nakamura Y, et al. HLA-DRB1*04:05 allele is associated with intracortical lesions on three-dimensional double inversion recovery images in Japanese patients with multiple sclerosis. *Multiple Sclerosis Journal*. 2018;24(6):710-720. doi:10.1177/1352458517707067

6. Masterman T, Ligers A, Olsson T, Andersson M, Olerup O, Hillert J. HLA-DR15 is associated with lower age at onset in multiple sclerosis. *Annals of Neurology*. 2000;48(2):211-219. doi:10.1002/1531-8249(200008)48:2<211::AID-ANA11>3.0.CO;2-R

7. Healy BC, Liguori M, Tran D, et al. HLA B*44: protective effects in MS susceptibility and MRI outcome measures. *Neurology*. 2010;75(7):634-640. doi:10.1212/WNL.0b013e3181ed9c9c

8. Isobe N, Keshavan A, Gourraud PA, et al. Association of HLA Genetic Risk Burden With Disease Phenotypes in Multiple Sclerosis. *JAMA Neurology*. 2016;73(7):795. doi:10.1001/jamaneurol.2016.0980

9. Liguori M, Healy BC, Glanz BI, et al. HLA (A-B-C and-DRB1) alleles and brain MRI changes in multiple sclerosis: A longitudinal study. *Genes and Immunity*. 2011;12(3):183-190. doi:10.1038/gene.2010.58

10. Pan G, Simpson S, Van Der Mei I, et al. Role of genetic susceptibility variants in predicting clinical course in multiple sclerosis: A cohort study. *Journal of Neurology, Neurosurgery and Psychiatry*. 2016;87(11):1204-1211. doi:10.1136/jnnp-2016-313722

11. Lysandropoulos AP, Mavroudakis N, Pandolfo M, et al. HLA genotype as a marker of multiple sclerosis prognosis: A pilot study. *Journal of the Neurological Sciences*. 2017;375:348-354. doi:10.1016/j.jns.2017.02.019

12. Misicka E, Sept C, Briggs FBS. Predicting onset of secondary-progressive multiple sclerosis using genetic and non-genetic factors. *Journal of Neurology*. 2020;267(8):2328-2339. doi:10.1007/s00415-020-09850-z

13. Baranzini SE, Wang J, Gibson RA, et al. Genome-wide association analysis of susceptibility and clinical phenotype in multiple sclerosis. *Hum Mol Genet*. 2009;18(4):767-778. doi:10.1093/hmg/ddn388

14. Qiu W, Raven S, James I, et al. Spinal cord involvement in multiple sclerosis: A correlative MRI and high-resolution HLA-DRB1 genotyping study. *Journal of the Neurological Sciences*. 2011;300(1-2):114-119. doi:10.1016/j.jns.2010.09.006

15. Kreft KL, van Meurs M, Wierenga-Wolf AF, et al. Abundant kif21b is associated with accelerated progression in neurodegenerative diseases. *Acta Neuropathologica Communications*. 2014;2(1):1-13. doi:10.1186/s40478-014-0144-4

16. Patsopoulos NA, Baranzini SE, Santaniello A, et al. Multiple sclerosis genomic map implicates peripheral immune cells and microglia in susceptibility. *Science (1979)*. 2019;365(6460). doi:10.1126/science.aav7188

17. Inkster B, Strijbis EMM, Vounou M, et al. Histone deacetylase gene variants predict brain volume changes in multiple sclerosis. *Neurobiology of Aging*. 2013;34(1):238-247. doi:10.1016/j.neurobiolaging.2012.07.007

18. Clarelli F, Assunta Rocca M, Santoro S, et al. Assessment of the genetic contribution to brain magnetic resonance imaging lesion load and atrophy measures in multiple sclerosis patients. *European Journal of Neurology*. 2021;28(8):2513-2522. doi:10.1111/ene.14872

19. Zhou Y, Graves JS, Simpson S, et al. Genetic variation in the gene LRP2 increases relapse risk in multiple sclerosis. *Journal of Neurology, Neurosurgery and Psychiatry*. 2017;88(10):864-868. doi:10.1136/jnnp-2017-315971

20. Hilven K, Vandebergh M, Smets I, Mallants K, Goris A, Dubois B. Genetic basis for relapse rate in multiple sclerosis: Association with LRP2 genetic variation. *Multiple Sclerosis Journal*. 2018;24(13):1773-1775. doi:10.1177/1352458517749894

21. Graves JS, Barcellos LF, Simpson S, et al. The multiple sclerosis risk allele within the AHI1 gene is associated with relapses in children and adults. *Mult Scler Relat Disord*. 2018;19(12):161-165. doi:10.1016/j.msard.2017.10.008

22. Lin R, Taylor B V., Simpson S, et al. Association between multiple sclerosis risk-associated SNPs and relapse and disability - A prospective cohort study. *Multiple Sclerosis Journal*. 2014;20(3):313-321. doi:10.1177/1352458513496882

23. Balnyte R, Rastenyte D, Vaitkus A, et al. The importance of HLA DRB1 gene allele to clinical features and disability in patients with multiple sclerosis in Lithuania. *BMC Neurology*. 2013;13(1):1. doi:10.1186/1471-2377-13-77

24. Balnytė R, Rastenytė D, Vaitkus A, Skrodenienė E, Vitkauskienė A, Ulozienė I. Associations of HLA DRB1 alleles with IgG oligoclonal bands and their influence on multiple sclerosis course and disability status. *Medicina (Lithuania)*. 2016;52(4):217-222. doi:10.1016/j.medici.2016.07.004

25. Schmidt S, Pericak-Vance MA, Sawcer S, et al. Allelic association of sequence variants in the herpes virus entry mediator-B gene (PVRL2) with the severity of multiple sclerosis. *Genes Immun*. 2006;7(5):384-392. doi:10.1038/sj.gene.6364311

26. Zhao S, Jing W, Samuels DC, Sheng Q, Shyr Y, Guo Y. Strategies for processing and quality control of Illumina genotyping arrays. *Brief Bioinform*. 2018;19(5):765-775. doi:10.1093/bib/bbx012
